# Late Gadolinium Enhancement Amount As an Independent Risk Factor for the Incidence of Adverse Cardiovascular Events in Patients with Stage C or D Heart Failure

**DOI:** 10.3389/fphys.2016.00484

**Published:** 2016-10-28

**Authors:** Tong Liu, Xiaohai Ma, Wei Liu, Shukuan Ling, Lei Zhao, Lei Xu, Deli Song, Jie Liu, Zhonghua Sun, Zhanming Fan, Taiyang Luo, Junping Kang, Xiaohui Liu, Jianzeng Dong

**Affiliations:** ^1^Department of Cardiology, Capital Medical University, Beijing Anzhen HospitalBeijing, China; ^2^Department of Radiology, Capital Medical University, Beijing Anzhen HospitalBeijing, China; ^3^State Key Lab of Space Medicine Fundamentals and Application, China Astronaut Research and Training CenterBeijing, China; ^4^Department of Vascular Surgery, Chinese PLA General HospitalBeijing, China; ^5^Department of Medical Radiation Sciences, School of Science, Curtin UniversityPerth, WA, Australia

**Keywords:** heart failure, magnetic resonance imaging, late gadolinium enhancement, prognosis, adverse cardiovascular events

## Abstract

**Background:** Myocardial fibrosis (MF) is a risk factor for poor prognosis in dilated cardiomyopathy (DCM). Late gadolinium enhancement (LGE) of the myocardium on cardiac magnetic resonance (CMR) represents MF. We examined whether the LGE amount increases the incidence of adverse cardiovascular events in patients with stage C or D heart failure (HF).

**Methods:** Eighty-four consecutive patients with stage C or D HF, either ischemic or non-ischemic, were enrolled. Comprehensive clinical and CMR evaluations were performed. All patients were followed up for a composite endpoint of cardiovascular death, heart transplantation, and cardiac resynchronization therapy with defibrillator (CRT-D).

**Results:** LGE was present in 79.7% of the end-stage HF patients. LGE distribution patterns were mid-wall, epi-myocardial, endo-myocardial, and the morphological patterns were patchy, transmural, and diffuse. During the average follow-up of 544 days, 13 (15.5%) patients had endpoint events: 7 patients cardiac death, 2 patients heart transplantation, and 4 patients underwent CRT-D implantation. On univariate analysis, LGE quantification on cardiac magnetic resonance, blood urine nitrogen, QRS duration on electrocardiogram, left ventricular end-diastolic diameter (LVEDD), and left ventricular end-diastolic volume (LVEDV) on CMR had the strongest associations with the composite endpoint events. However, on multivariate analysis for both Model I (after adjusting for age, sex, and body mass index) and Model II (after adjusting for age, sex, BMI, renal function, QRS duration, and atrial fibrillation on electrocardiogram, the etiology of HF, LVEF, CMR-LVEDD, and CMR-LVEDV), LGE amount was a significant risk factor for composite endpoint events (Model I 6SD HR 1.037, 95%CI 1.005–1.071, *p* = 0.022; Model II 6SD HR 1.045, 95%CI 1.001–1.084, *p* = 0.022).

**Conclusion:** LGE amount from high-scale threshold on CMR increased the incidence of adverse cardiovascular events for patients in either stage C or D HF.

## Introduction

End-stage heart failure is associated with significant morbidity and mortality (Adams et al., 1996; Bart et al., 1997). To improve the prognosis, the optimal therapeutic strategies for patients with end-stage heart failure currently include cardioverter defibrillator implantation (ICD), cardiac resynchronization therapy (CRT), ventricular assist devices implantation (Anand et al., 2015), and heart transplantation (Yancy et al., 2013). Complex invasive therapeutic decisions for patients with stage C or D heart failure are mainly guided by left ventricular ejection fraction (LVEF; Epstein et al., 2013; Yancy et al., 2013). However, using LVEF as a parameter to identify eligible patients has a number of limitations (Gulati et al., 2013). Identifying the optimal prognosis factors for patients with end-stage heart failure remains a challenge.

The most common cause of end-stage heart failure includes ischemia-dilated cardiomyopathy (IDCM) and non-ischemia-dilated cardiomyopathy (NIDCM; Sisakian, 2014). Histologically, the main feature of end-stage damaged myocardium, whether ischemic or non-ischemic, is the development of interstitial and replacement fibrosis that varies in severity (Schaper et al., 1991; Yarbrough et al., 2012). Expansion of the extracellular matrix, which is observed in individuals with myocardial fibrosis, is associated with contractile impairment (Baig et al., 1998; Mann and Bristow, 2005) and provides a substrate for ventricular reentrant arrhythmia (Hsia and Marchlinski, 2002; Iles et al., 2011), which represents a strong risk factor for adverse cardiac outcomes (Assomull et al., 2006). Late gadolinium enhancement from cardiac magnetic resonance (LGE-CMR) is capable of detecting tissue abnormalities, particularly myocardial fibrosis (Nanjo et al., 2009). Researchers recently discovered that, in addition to LVEF, late gadolinium enhancement (LGE) could be used as a marker of poor prognosis in patients with NIDCM (Gulati et al., 2013; Neilan et al., 2013; Kuruvilla et al., 2014; Perazzolo Marra et al., 2014; Pöyhönen et al., 2014). However, little is known about the role of LGE quantification for patients with stage C or D heart failure; in addition, controversy persists regarding LGE amount and its prognostic value (Neilan et al., 2013; Perazzolo Marra et al., 2014).

Here, we aimed to assess the association between LGE-CMR quantification and the prognosis of patients with stage C or D heart failure. We hypothesized that LGE-CMR amount is associated with a higher risk of adverse cardiac outcomes in patients with stage C or D heart failure regardless of ischemic or non-ischemic etiology.

## Materials and methods

The Department of Heart Failure Program at Anzhen Hospital (Capital Medical University of Beijing, China) performed a prospective observational cohort study. The study enrolled 84 consecutive heart failure patients between January 1st, 2014 and December 31st, 2014, with ischemic or non-ischemic heart disease as defined by the World Health Organization/International Society and the Federation of Cardiology criteria (Richardson et al., 1996). Inclusion criteria included: stage C or D heart failure (Jessup et al., 2009; Chen-Scarabelli et al., 2015), ischemic or non-ischemic cardiomyopathy (IDCM and NIDCM), LVEF < 40%, and left ventricular end-diastolic diameter (LVEDD) >60 mm on echocardiography. Patients with valvular heart disease, congenital heart disease, infiltrative cardiomyopathy (i.e., sarcoidosis and amyloidosis), renal failure, acute myocardial infarction (AMI) within 1 month, poor compliance, or a history of CRT and ICD implantation were excluded from the study. All patients enrolled in the study underwent a comprehensive clinical evaluation, coronary angiography, and MRI. Ischemic dilated cardiomyopathy was diagnosed when coronary angiography showed >50% narrowing of the coronary artery lumen or when the patient had a history of MI. Non-ischemic dilated cardiomyopathy may have been idiopathic, familial/genetic, viral, immune, or alcoholic/toxic (Richardson et al., 1996). Patients enrolled in the study provided written informed consent. The protocol was approved by the Human Subjects Review Committee at Anzhen Hospital (Approval No. 2013007X). All of the experiments were performed in accordance with relevant guidelines and regulations.

All cardiac magnetic resonance (CMR) examinations were performed using a 3.0-T Magnetic Resonance Imaging (MRI) system (Verio, Siemens Medical Solutions, Erlangen, Germany) with a 32-channel phased-array coil. True fast imaging with steady state free precession breath-hold cine images were acquired in the short-axis, and 2-, 3-, and 4-chamber views encompassing the entire left ventricle (LV) volume from the apex to the base. Diastolic phase myocardial delayed enhancement images in the same orientations as in the cine images were acquired 10 min after intravenous infusion with gadolinium chelate contrast agent (Magnevist, Bayer Schering, Germany, 0.2 mmol/kg) with a prospectively ECG-gated gradient echo sequence with an inversion prepulse. Inversion times were optimized to null normal myocardium. Imaging parameters were as follows: repetition time/echo time, 4.1/1.6 ms; flip angle, 20°; image matrix, 256 × 130; section thickness, 8 mm (contiguous short axis) or 5 mm (long axis images), with no intersection gap.

The QMASS commercial software package (Medis, Netherland) was used to analyze the CMR DICOM images. The cardiac functional indexes, specifically the left ventricular ejection fraction (LVEF), volumes, and mass were quantified from cine images by using standard methods. The volume and mass of the LV was normalized to the body surface area. Two experienced observers, blinded to the clinical outcome independently determined the dichotomous presence or absence of LGE by reviewing all short and long axis contrast-enhanced images; regions of elevated signal intensity had to be confirmed in two spatial orientations. Once the LGE was present, the distribution of the LGE was recorded in the 17 segments according to the standards of the American Heart Association (AHA). Furthermore, the quantity of hyper-enhancement was defined as regions with an abnormally increased signal intensity >2SD and >6SD of the peak remote. According to other studies (Flett et al., 2011; Maron, 2013; Moravsky et al., 2013; Chan et al., 2014) as well as the characteristics of our study population, the high gray-scale threshold (6SD) as the LGE quantification method was mainly used here. For each short axis cross-section, after the endocardial and epi-cardial borders were traced, a region of interest (ROI) averaging 50 mm^2^ was defined within the normal remote myocardium, in an area with uniform myocardial suppression, free of artifacts. The commercial software automatically calculated the %Myo (LGE positive volume/Total myocardium volume × 100%).

Follow-up was performed until January of 2016. The patients were followed-up for major adverse cardiac events (MACE), including cardiovascular death, cardiac transplantation, and cardiac resynchronization therapy with defibrillator (CRT-D). CRT-D implantation was based on information obtained from medical records and telephone calls. CRT-Ds were only implanted in patients who fit the guideline recommendation criteria for CRT-Ds and who had recurrent admissions for heart failure, despite undergoing at least 6 months of optimal medical therapy (Yancy et al., 2013).

Continuous variables are expressed as mean ± standard deviations (*SD*), while categorical variables are expressed as a frequency (%). Continuous variables were compared using an unpaired Student *t*-test or a Mann-Whitney nonparametric test, as appropriate. Categorical variables were performed using the Pearson Chi-Square test and the Fisher exact test. A *P* < 0.05 was deemed statistically significant and all statistical tests were 2-sided. Receiver operating characteristic (ROC) curves constructed using Bootstrap resampling (times = 500) were used to find the optimal cut-off values for LGE quantification (with maximizing the sum of sensitivity and specificity) as measured by 6SD methods to predict MACE. Survival estimates and cumulative event rates were compared by the Kaplan–Meier method by using the time-to-first event for each endpoint. The log-rank test was used to compare the Kaplan–Meier survival curves. Cox regression analyses were used to estimate the hazard ratio (HR) for adverse events and their corresponding 95% confidence intervals (CI). Univariate Cox regression analyses were used to examine all significant variables. We also adjusted for variables that, when added to this model, changed the Matched Hazard Ratio by at least 10%. All analyses were performed using Empower (R) (http://www.empowerstats.com, X&Y solutions, Inc., Boston MA) and R (http://www.R-project.org).

## Results

Of the 84 patients with either ischemic or non-ischemic heart failure, the average age was 52 years (51.5 ± 12.3) at the time of CMR imaging, in addition, 65 of the these patients (77.4%) were male. The mean LVEF on CMR was 16.4 ± 8.8%, and all patients at presentation had either stage C or stage D heart failure. After extensive cardiac examination of all patients at baseline, 57 patients (67.9%) were diagnosed with non-ischemic dilated cardiomyopathy, and 27 patients (32.1%) were diagnosed with ischemic dilated cardiomyopathy, and atrial fibrillation was observed on the electrocardiogram among 20.5% of patients with heart failure, Table 1. Among the entire cohort, 95% were prescribed either an angiotensin-converting enzyme inhibitor or an angiotensin II receptor blocker, 95% were prescribed a beta-blocker, 94% were prescribed an aldosterone antagonist, until then, 96.5% were prescribed loop diuretics.

**Table 1:** **Baseline characteristics of patients with stage C&D heart failure**.

	**All patients (*n* = 84)**	**Event-free patients (*n* = 71)**	**Event patients (*n* = 13)**	***P***
Age, mean (*SD*) (years)	51.5 ± 12.3	50.6 ± 12.4	56.8 ± 10.5	0.096
Male (%)	65 (77.38%)	54 (76.1%)	11 (84.6%)	0.498
BMI, mean (*SD*) (kg/m^2^)	26.7 ± 4.3	26.8 ± 4.2	25.8 ± 4.6	0.450
Etiology of heart failure				0.596
IDCM (%)	27 (32.1%)	22 (31.0%)	5 (38.5%)	
NIDCM (%)	57 (67.9%)	49 (69.0%)	8 (61.5%)	
SBP, mean (*SD*) (mmHg)	113.1 ± 15.08	114.4 ± 14.9	106.2 ± 14.7	0.069
Atrial fibrillation (%)	17 (20.5%)	12 (17.1%)	5 (38.5%)	0.08
hs-CRP mean (*SD*) (mg/L)	6.7 ± 8.6	6.4 ± 8.4	7.9 ± 9.7	0.565
BUN mean (*SD*) (mmol/L)	7.4 ± 1.9	7.2 ± 1.7	8.7 ± 2.7	0.009
**MEDICATION**				
ACEI/ARB (%)	80 (95.2%)	69 (97.2%)	11 (84.6%)	0.875
Beta-blocker (%)	80 (95.2%)	68 (95.8%)	12 (92.3%)	0.589
Spironolactone (%)	79 (94.1%)	68 (95.8%)	11 (84.6%)	0.118
Diuretics (%)	81 (96.4%)	69 (97.2%)	12 (92.3%)	0.384
QRS duration mean (*SD*) (mm)	137.2 ± 36.0	132.5 ± 31.3	162.5 ± 49.2	0.005
**CMR CHARACTERISTICS**				
CMR-LVEDD, mean (*SD*) (mm)	70.9 ± 9.7	70.2 ± 8.8	75.0 ± 13.3	0.101
CMR-EF, mean (*SD*)	16.4 ± 8.8	17.0 ± 8.8	13.2 ± 8.5	0.160
CMR-LVEDV, mean (*SD*) (ml)	279.8 ± 103.9	265.5 ± 84.7	357.8 ± 158.1	0.003
LGE 2SD%Myo, mean (*SD*)	40.3 ± 21.1	36.9 ± 18.9	58.5 ± 23.6	<0.001
LGE 6SD%Myo, mean (*SD*)	8.5 ± 14.1	6.1 ± 10.6	21.6 ± 22.2	<0.001

*IDCM, ischemic dilated cardiomyopathy; NIDCM, non-ischemic dilated cardiomyopathy; BMI, Body mass index; SBP, systolic blood pressure; hs-CRP, hypersensitive C-reactive protein; BUN, blood urea nitrogen; ACEI/ARB, angiotensin-converting enzyme inhibitor or an angiotensin II receptor blocker; LVEDD, left ventricular end-diastolic diameter, EF ejection fraction; LVEDV, left ventricular end diastolic volume; SD, standard deviation*.

**Figure 1:**
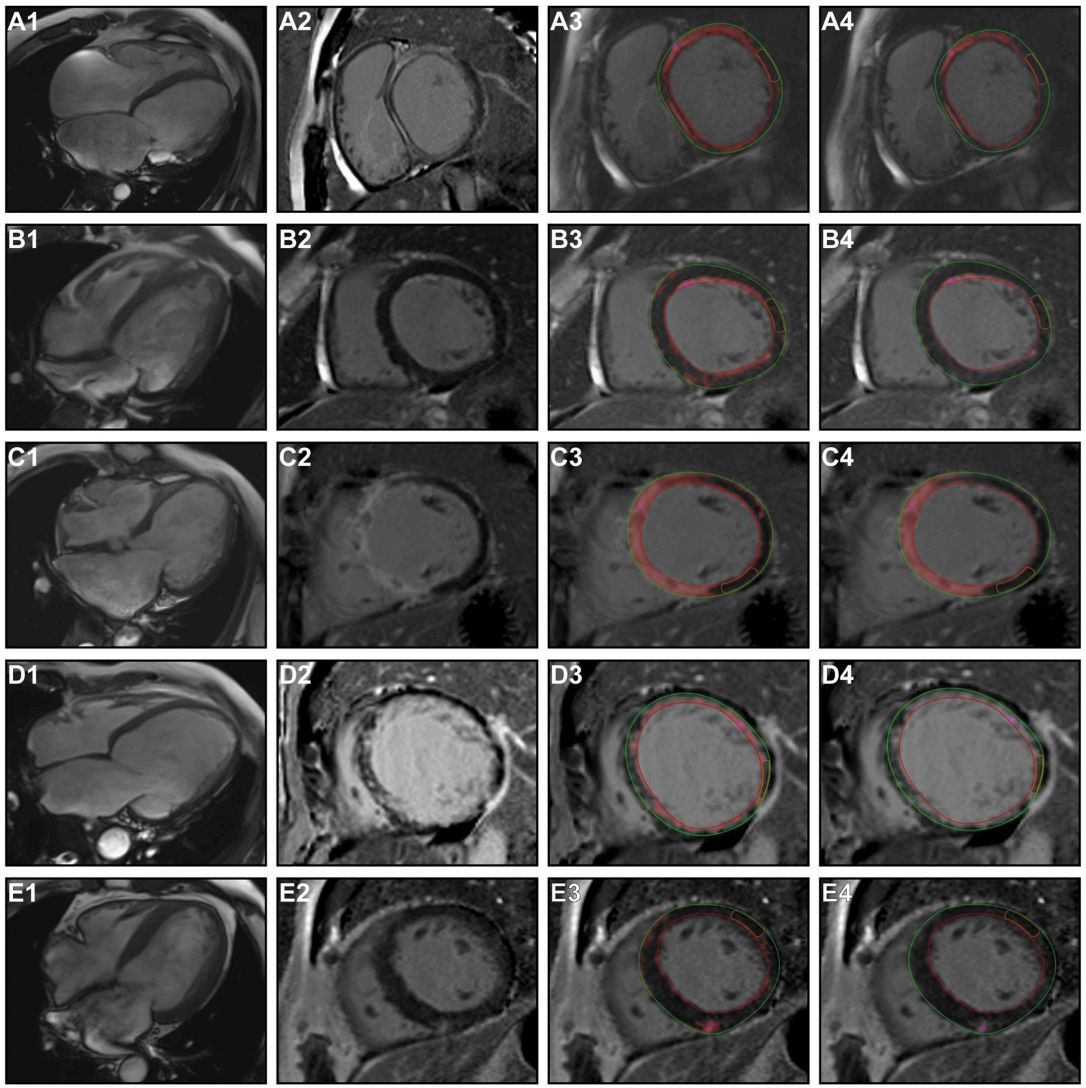
**Different patterns and distributions of LGE in patients with end-stage heart failure**. The first panel shows the four-chamber view of the heart, the second panel displayed the patterns of LGE. The 2SD and 6SD algorithm quantitative assessments of myocardial fibrosis are marked in the third and fourth panels. Rows A through E depict mid-wall, endo-myocardial, transmural, diffuse, and patchy enhancements in the short axis. LGE, late gadolinium enhancement; *SD*, standard deviation.

**Figure 2:**
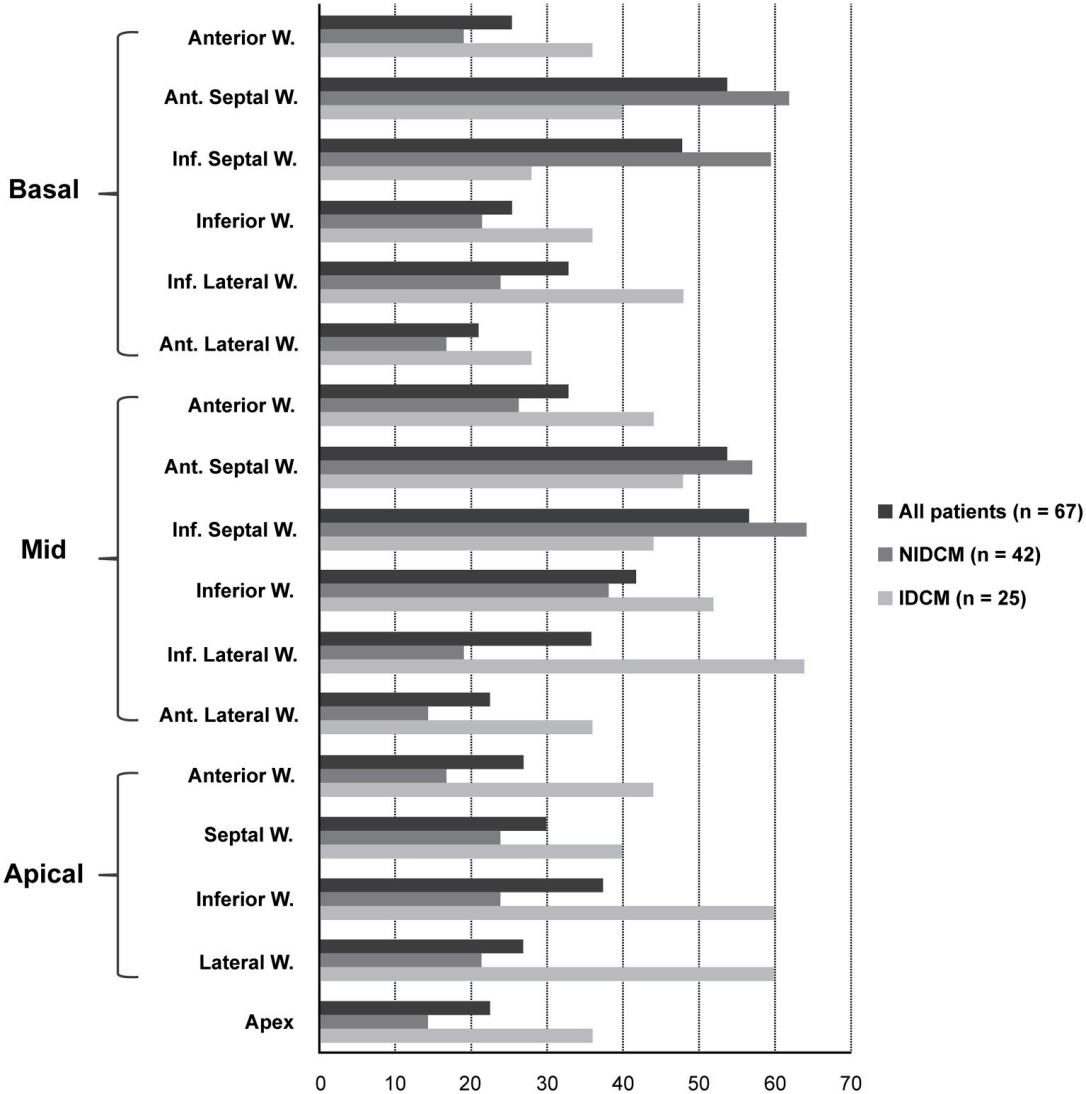
**The distribution of LGE in individual myocardial segments in all LGE positive patients (blue bars), in patients with IDCM (green bars), and in patients with NIDCM (red bars)**. The diagram demonstrates that LGE in patients with NIDCM occurs most commonly in the septal wall of the left ventricle while LGE in patients with IDCM commonly involves the coronary artery dominated area. IDCM, ischemia-dilated cardiomyopathy; LGE, late gadolinium enhancement; NICD, non-ischemia-dilated cardiomyopathy.

**Figure 3:**
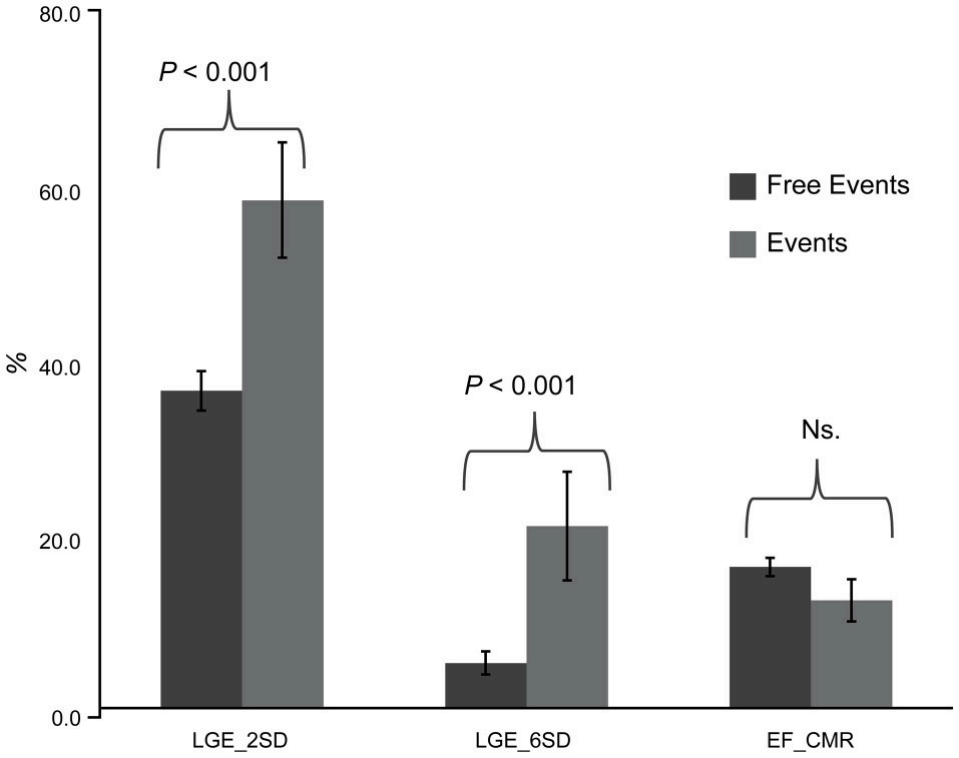
**The diagram displays statistical differences in %Myo of LGE for both 2-***SD*** and 6-***SD*** algorithms (mean ± SE, ***P*** < 0.001) in patients with and without events**. No statistical difference in conventional cardiac ejection function index (mean ± SE, *P* = 0.16) is detected. LGE, late gadolinium enhancement; *SD*, standard deviation.

LGE was present in 67 patients (79.76%). LGE distribution pattern was mid-wall and epi-myocardial in 24 (35.8%, 24/67) patients, endo-myocardium in 12 (17.9%, 12/67) patients, patchy in 6 (9.0%, 6/67) patients, transmural in 20 (29.9%, 20/67) patients, and diffuse in 5 (7.5%, 5/67) patients (Figure 1). The involved myocardial segments listed in the Figure 2, based on the all study patients discriminated with the ischemic and non-ischemic etiology (IDCM and NIDCM) of heart failure. The average volume of LGE, expressed as a percentage of the total LV volume by using the 2SD & 6SD methods, was significantly greater in patients with events than in those with no events (LGE 2SD %Myo: 58.5 ± 6.55 vs. 36.9 ± 2.24; LGE 6SD %Myo: 21.6 ± 6.16 vs. 6.1 ± 1.26 in events patients vs. free-event patients, respectively; *p* < 0.001). However, although the LVEF on CMR was lower in patients with events, these differences were not statistically significant (13.2 ± 2.36 vs. 17.0 ± 1.04% *p* = 0.16; Figure 3).

During the average 544 days follow-up period (min 365 days and max 673 days), 13 (15.5%) patients developed major adverse cardiac events; in addition, 7 patients died, 2 patients underwent cardiac transplantation, and 4 patients underwent CRT-D implantation.

The results of the univariate analyses of major adverse events during the follow-up period are summarized in Table 2. Univariate analyses showed that the widened QRS duration on EKG, and the renal function, the amount of LV-LGE, LVEDD, and left ventricular end-diastolic volume (LVEDV) on CMR were associated with significant increases in the incidence of adverse cardiovascular events. However, the predicted value of LVEF on CMR was not significantly different (HR 0.956; 95%CI 0.878–1.041; *P* = 0.305). In the multivariable analysis shown in Table 3, the amount of LV-LGE was the only independent risk factor for major adverse events in Model I (HR_adj_ 1.44/10% increase in 6SD %Myo, 95%CI 1.05–1.98, *P* = 0.022) after adjusting for the patients' age, sex, and body mass index (BMI), and in Model II (HR_adj_ 1.54/10% increase in 6SD %Myo, 95%CI 1.06–2.24, *P* = 0.022) after adjusting for patients' age, BMI, sex, blood urea nitrogen (BUN), QRS duration, and Atrial fibrillation (%) on EKG, etiology of heart failure, LVEF, CMR- LVEDD, and LVEDV.

**Table 2:** **Univariate analysis for major adverse events in patients with stage C or stage D heart failure during follow-up**.

**Variable**	**No. of event/No. of participants (%)**	**HR**	**95% CI**	***P*-value**
Age (years)	13/84 (15.47)	1.043	0.995–1.094	0.082
**SEX**				
Female	2/19 (10.52)	Ref	–	–
Male	11/65 (16.92)	2.359	0.503–11.058	0.276
BMI (kg/m^2^)	13/84 (15.47)	0.970	0.852–1.104	0.647
**ETIOLOGY OF HF**				
IDCM	5/27 (18.51)	Ref	–	–
NIDCM	8/57 (14.04)	0.823	0.267–2.534	0.734
**HEART RHYTHM**				
Sinus rhythm	8/66 (12.12)	Ref	–	–
Atrial fibrillation	5/17 (29.41)	2.145	0.678–6.789	0.194
BUN (mmol/L)	13/84 (15.47)	1.344	1.068–1.692	0.012
QRS duration (mm)	13/84 (15.47)	1.015	1.001–1.029	0.039
CMR-LVEDD (mm)	13/84 (15.47)	1.078	1.023–1.135	0.005
CMR-LVEDV (ml)	13/84 (15.47)	1.006	1.002–1.010	0.002
CMR-LVEF	13/84 (15.47)	0.956	0.878–1.041	0.305
2SD %Myo	13/84 (15.47)	1.034	1.011–1.057	0.003
6SD %Myo	13/84 (15.47)	1.028	1.005–1.051	0.016

*BMI, body mass index; BUN, blood urine nitrogen; CI, confidence interval; CMR-LVEDD, cardiac magnetic resonance left ventricular end-diastolic diameter; CMR-LVEDV, cardiac magnetic resonance left ventricular end-diastolic volume; HR, hazard ratio; IDCM, ischemic cardiomyopathy; NIDCM, non-ischemic cardiomyopathy; SD, standard deviation, %Myo, LGE positive volume/Total myocardium volume × 100%*.

**Table 3:** **Multivariate analysis with LGE-CMR quantification for major adverse events in patients with Stage C or D heart failure during follow-up**.

	**Model I**			**Model II**		
	**HR**	**95% CI**	***P*-value**	**HR**	**95% CI**	***P*-value**
6SD %Myo per 1% increase	1.037	1.005–1.071	0.022	1.045	1.001–1.084	0.022
6SD %Myo per 10% increase	1.44	1.053–1.982	0.022	1.546	1.065–2.244	0.022

*Model I adjust for: age, sex, BMI. Model II adjust for: age, sex, BMI, BUN, QRS duration on EKG, Atrial fibrillation (%), etiology of heart failure, LVEF, CMR-LVEDD, CMR-LVEDV. SD, standard deviation; HR, hazard ratio; CI, confidence interval*.

**Figure 4:**
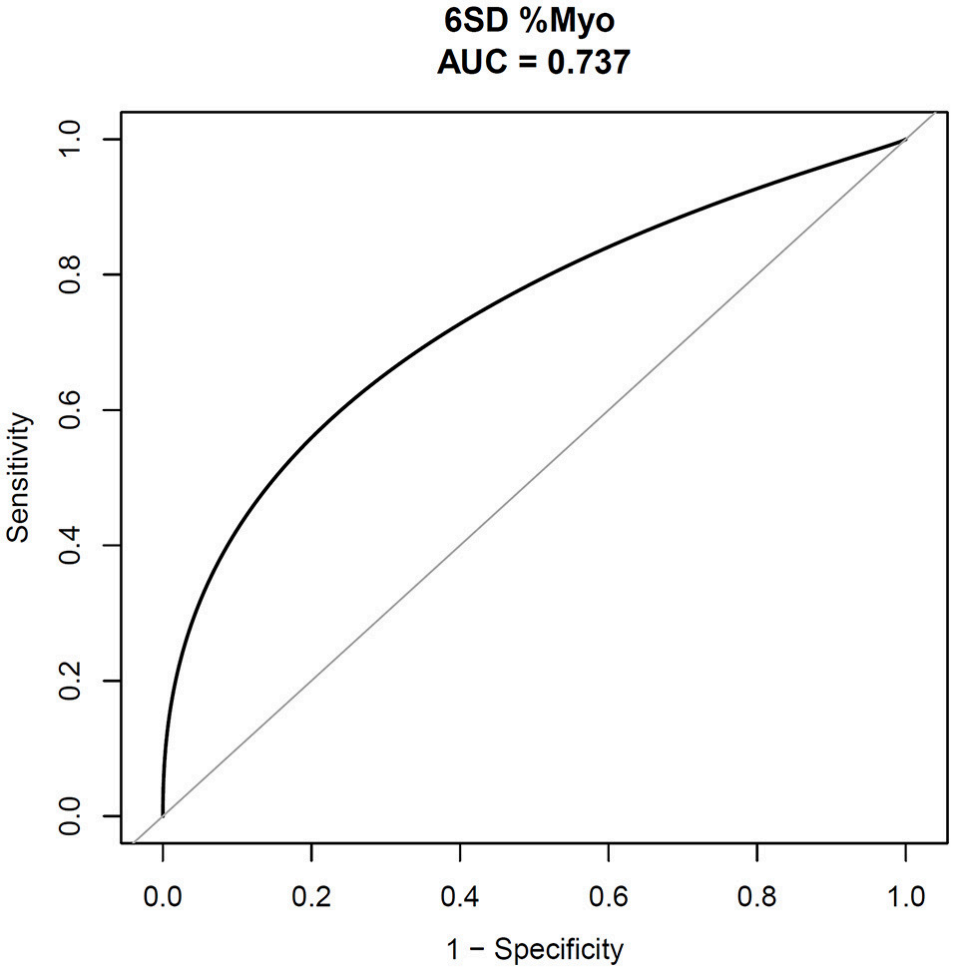
**ROC curves for the amount of LGE, using Bootstrap resampling (times = 500) for the association of major adverse cardiac events**. AUC confidence interval and significance tests using Bootstrap resampling. The best cut point was based on maximizing the sum of sensitivity and specificity. The analysis reveals that the percentage of LGE by volume of >10.91%, using the 6-SD method (AUC: 0.74, 95% CI 0.58–0.89; sensitivity: 61.5%; specificity: 85.9%). ROC, receiver-operating characteristic; AUC, area under the curve; *SD*, standard deviation; CI, confidence interval.

**Figure 5:**
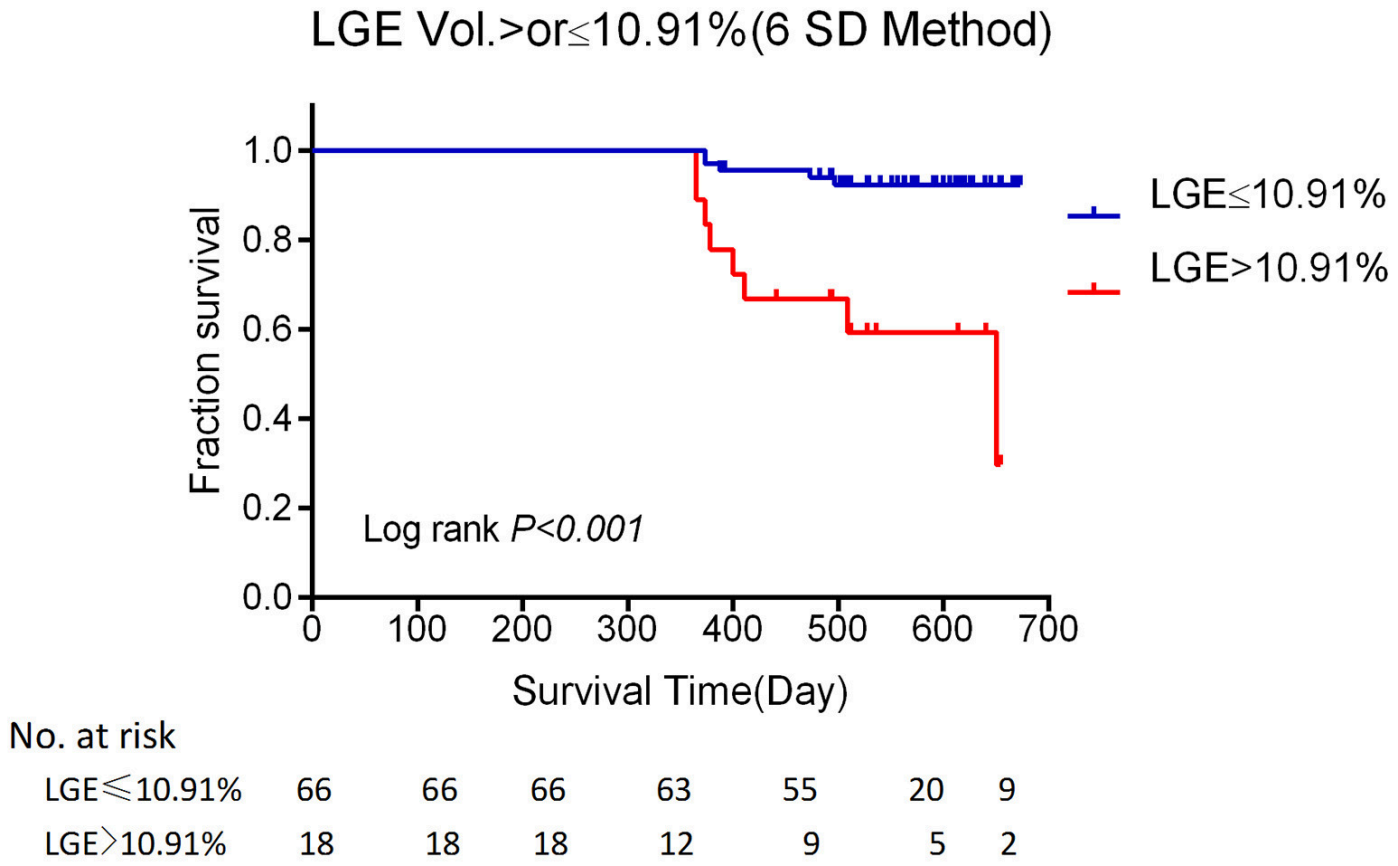
**Kaplan–Meier event-free survival curve**. Kaplan–Meier analysis of freedom from major adverse cardiac events according to: LGE of >10.91 or < 10.91% of the volume of the left ventricle, as measured using the 6SD method. (Log-rank, *P* < 0.001). LGE, late gadolinium enhancement; *SD*, standard deviation.

ROC curves of LGE quantification were used to determine the optimal cut-off values for prediction of events during follow-up by using a bootstrap resampling (times = 500) showed an area under curve of 0.74 (95%CI 0.58–0.89; Figure 4). The best cut point based on maximizing the sum of sensitivity and specificity for 6SD method was 10.91% (sensitivity: 61.5%; specificity: 85.9%). Kaplan–Meier survival analysis (Figure 5) showed a significant difference between patients stratified by the amount of LGE, that the extent of LGE of >10.91 or < 10.91% of the volume of the left ventricle, as measured using the 6SD method (Log-rank, *P* < 0.001).

## Discussion

Heart failure is a serious condition that constitutes medical and financial burdens in developed and developing countries. Approximately half of all patients with heart failure die within 5 years after receiving the diagnosis, which is worse than that for many cancers (Katz, 1990). The most common cause of clinical heart failure is dilated cardiomyopathy, which is ischemic or non-ischemic in etiology, and associated with high morbidity and mortality (Hunt et al., 2005; Maron et al., 2006).

Histologically, the end-stage damaged myocardium is characterized by a diverse abnormal myocardial substrate including reparative (replacement) and reactive (interstitial and perivascular) fibrosis, leading to expanded extracellular matrix (de Leeuw et al., 2001). LGE can be used to non-invasively identify of myocardial fibrosis. Researchers also discovered that LGE could be used as a marker of poor prognosis in patients with NIDCM (Gulati et al., 2013; Neilan et al., 2013; Kuruvilla et al., 2014; Perazzolo Marra et al., 2014) and HCM (Chan et al., 2014). However, the current LGE cannot differentiate between interstitial and replacement fibrosis (Moravsky et al., 2013), and consensus is lacking about how to quantify LGE in patients with stage C or D heart failure. There are several quantitative techniques for LGE, including a gray-scale threshold a number of *SD*s above the mean signal intensity within a remote region containing normal “nulled” myocardium (i.e., 2, 4, 5, or 6SD) and the full width at half maximum (FWHM) method (Flett et al., 2011; Maron, 2013; Moravsky et al., 2013; Contaldi et al., 2016). Moravsky et al. (2013) first provided an evaluation correlating histological findings with a variety of LGE quantification methods in patients with HCM. Their study discovered (Maron, 2013) that a high gray-scale threshold provided a better representation of total fibrosis burden (interstitial and replacement fibrosis) than the lower thresholds and the FWHM method. Considering the diverse histopathological abnormalities including interstitial and replacement fibrosis in end-stage heart failure patients (de Leeuw et al., 2001), the high gray-scale threshold (6SD) as the LGE quantification method was used here. Our study was designed to evaluate the correlation between the amount of LGE (6SD) on CMR and major adverse events in a large population of patients with stage C or D heart failure and dilated cardiomyopathy despite ischemic or non-ischemic etiology.

The major finding of our study was that the amount of LGE from high-scale threshold on CMR increased the likelihood for adverse cardiovascular outcomes in patients with stage C or stage D heart failure. This association was independent of the LVEF and the etiology of heart failure (IDCM and NIDCM; Tables 2, 3). Using multivariable Cox regression analysis, we determined that the adjusted HRs for patients with stage C or stage D heart failure with 6SD %Myo were 1.037 (*P* = 0.022), in Model I, and 1.045 (*P* = 0.022) in Model II for cardiovascular death and cardiac transplantation or implanted CRT-D. Our results also indicated that LGE cutoff values of 10.91% (6SD method) for patients with stage C or stage D heart failure are associated with poor outcomes.

The CMR LGE patterns and distributions in our cohort study are consistent with those reported in prior pathologic studies of NIDCM (Assomull et al., 2006; Wu et al., 2008; Gulati et al., 2013), which involved the mid-myocardium of the septal wall (Figure 2). However, the delayed enhancement in IDCM tends to occur in the endocardium or transmural region and follows a vascular distribution. The pathophysiology underlying CMR abnormalities is uncertain since dilated cardiomyopathy is the final common pathway for presumably multiple etiologies. Myocardial fibrosis in end-stage heart failure is due to activation of the circulating renin-angiotensin system (RAS) and aldosterone production that eventually lead to progressive myocyte dysfunction, apoptosis, and fibroblast hyperplasia (Swynghedauw, 1999).

Perazzolo Marra et al. (2014) evaluated the impact of myocardial fibrosis presence and amount on NIDCM prediction. Their study showed that LV-LGE was a powerful and independent predictor of prognosis, while the amount of LGE and its distribution did not provide additional prognostic value. Conversely, in our cohort study, there was a strong association between the amount of LGE and poor prognosis. This difference may be due to differences in the study population. In our study, all patients had obvious symptoms of stage C or D heart failure. Myocardial fibrosis is common in these patients. Accordingly, the percentage of patients with LGE (79.76%) in our study was significantly higher than reported by previous studies (Perazzolo Marra et al., 2014). Although the current LGE cannot differentiate interstitial and replacement fibrosis (Moravsky et al., 2013), it can provide a total fibrosis burden (interstitial and replacement fibrosis; Maron, 2013). This kind of histopathological abnormality would lead to an expanded extracellular matrix (de Leeuw et al., 2001). Chan et al. (2014) found that extent of LGE was associated with an increased risk of events (adjusted hazard ratio, 1.46/10% increase in LGE; *P* = 0.002). Although the study populations were very different, the results of patients with hypertrophic cardiomyopathy (HCM) in their study and those with stage C or D heart failure in our study were consistent with the results reported by Chan et al. (2014). Our data also showed that extensive LGE remained an important marker of an increased risk of major adverse events (HRadj, 1.54/10% increase in 6SD %Myo, *P* = 0.022), even after the adjustment for other relevant variables, including LVEF. The Bootstrap resampling (times = 500) was used to find the optimal cut-off value and the 6SD LGE >10.91% of LV mass conferred the high risk of major adverse events in patients with stage C or D heart failure.

The current assessment of the prognosis of patients with stage C or D heart failure, either ischemic or non-ischemic in etiology, is purely based on a decreased LVEF (Rihal et al., 1994; Grzybowski et al., 1996), which has been considered a strong risk factor for mortality. However, although the mortality in patients with stage C or D heart failure increases as LVEF decreases, the association is very weak in patients with severe systolic dysfunction (Dec and Fuster, 1994). Clinical trials have provided evidence that LVEF and LVEDD can be reversed by medical therapy (Aleksova et al., 2009). Therefore, in patients with severe left ventricular systolic impairment, a prognosis evaluation based on LVEF alone is insufficient. In the present study, MF quantification provided additional prognostic value across the entire range of LVEF for all end points.

In conclusion, the detection of myocardial fibrosis by measuring LGE from high-scale threshold on CMR increased poor outcomes for primary endpoints in patients with stage C or D heart failure with either ischemic or non-ischemic in etiology. These findings have potentially important implications which may have applications in the reclassification of patient groups suitable for device therapy (i.e., CRT-D, transplantation, left ventricular assist device implantation) with end-stage heart failure. Further research efforts should include a prospectively enrolled cohort to determine whether CMR in combination with other novel markers could help identify patients with stage C or D heart failure who may benefit from additional device treatment.

## Limitations

The major limitation of our study is the relatively small cohort size and short follow-up period. Furthermore, there were only two cases of heart transplantations in our study. The pathological report for the recipient's heart further confirmed our diagnosis of IDCM and NIDCM. We will continue to follow the study population to collect additional pathological results in support of our initial diagnosis. Finally, we quantified LGE using a signal intensity threshold of 6SD above a remote reference region. The traditional LGE measurement method cannot detect small patches as well as diffuse microscopic fibrosis. The T1 mapping technique may enable us to improve the clinical assessment of diffuse myocardial fibrosis.

## Author contributions

TL and XM conceived and designed the experiments, and wrote the manuscript together. They have contributed equally to the work. TL and JD are responsible for the data and result. WL, DS, TYL, JK, and XL performed the experiment. SL and JL analyzed the data. LZ and ZF provide reagents and analysis tools. ZS and LX helped to revise the manuscript.

## Funding

This study was supported by the China Natural Science Funding (81101173 and 81470429) and the High Levels of Health Technical Personnel in Beijing City Health System (2013-3-005). The funders had no role in study design, data collection and analysis, decision to publish, or preparation of the manuscript.

### Conflict of interest statement

The authors declare that the research was conducted in the absence of any commercial or financial relationships that could be construed as a potential conflict of interest.
